# Practical Surgery, Including Ligations and Amputations

**Published:** 1879-04

**Authors:** 


*Practical Surgery, including Ligations and Amputations.* By J. EWING MEARS, M. D. With two hundred and twenty-seven illustrations. Philadelphia: Lindsay and Blakiston, 1878.

This is a manual of Minor Surgery, giving in brief form, with ample and well chosen illustration: Part I—Surgical Dressings; Part II—Bandaging; Part III—Ligations; Part IV—Amputations. These subjects are considered in practical and instructive manner, and will prove a judicious and safe guide to the practitioner.

